# Clinical–genomic risk discordance and its impact on treatment selection in premenopausal women with node-positive hormone receptor-positive breast cancer

**DOI:** 10.1186/s12885-026-15913-7

**Published:** 2026-04-06

**Authors:** Mariko Nishikawa, Shinichiro Kashiwagi, Asuka Kochi, Chika Watanabe, Koji Takada, Yukie Tauchi, Kana Ogisawa, Haruhito Kinoshita, Masatsune Shibutani, Tamami Morisaki

**Affiliations:** 1 Department of Breast Surgical Oncology, Osaka Metropolitan University, Graduate School of Medicine, 1-4-3 Asahi-machi, Abeno-ku, Osaka, 545-8585 Japan; 2 Department of Gastrointestinal Surgery, Osaka Metropolitan University, Graduate School of Medicine, 1-4-3 Asahi-machi, Abeno-ku, Osaka, 545-8585 Japan

**Keywords:** Clinical–genomic risk discordance, Oncotype DX, Premenopausal breast cancer, Node-positive disease, Adjuvant treatment selection, Endocrine therapy

## Abstract

**Purpose:**

The 21-gene Oncotype DX Recurrence Score (RS) is widely used to guide adjuvant chemotherapy decisions in hormone receptor-positive (HR+), human epidermal growth factor receptor 2-negative (HER2-) breast cancer. However, the clinical interpretation of RS remains challenging in premenopausal women with node-positive disease, where discordance between genomic and clinicopathological risk is frequently encountered. This study aimed to elucidate the determinants of clinical–genomic risk discordance and to clarify its impact on real-world treatment selection, with a specific focus on premenopausal node-positive patients.

**Methods:**

We retrospectively analyzed 290 patients with HR+/HER2- breast cancer who underwent curative surgery and Oncotype DX testing between September 2023 and November 2025. Clinical risk was defined according to the St. Gallen criteria, and concordance or discordance was determined based on agreement or disagreement between clinical risk and RS. Logistic regression analyses were performed to identify predictors of discordance in the overall cohort and in node-positive patients. Treatment allocation was further examined in a predefined subgroup of 28 premenopausal patients with node-positive disease. Treatment strategies were categorized as chemotherapy plus endocrine therapy, endocrine therapy combined with systemic intensification (abemaciclib or S-1), or endocrine therapy alone.

**Results:**

Clinical–genomic risk discordance was observed in 123 patients (42.4%). In multivariable analysis of the overall cohort, larger tumor size (OR 8.33) and nodal positivity (OR 6.43) were independently associated with discordance, indicating a strong association with tumor burden. In contrast, among node-positive patients, premenopausal status (OR 0.18, *p* = 0.045) and high proliferative activity (Ki-67 > 20%; OR 4.81, *p* = 0.048) were independently associated with discordance, indicating a distinct biological pattern associated with proliferative activity in premenopausal disease. In the premenopausal node-positive subgroup, all concordant cases received intravenous chemotherapy, whereas discordant cases showed marked heterogeneity in treatment selection: chemotherapy in 2 patients (8.0%), endocrine therapy combined with targeted or fluoropyrimidine-based intensification strategies (including abemaciclib or S-1) in 11 patients (44.0%), and endocrine therapy alone in 12 patients (*p <* 0.001).

**Conclusions:**

Clinical–genomic risk discordance is frequent in premenopausal women with node-positive hormone receptor-positive, HER2-negative breast cancer and is associated with distinct biological characteristics, including high proliferative activity. In real-world clinical practice, discordance was linked to substantial variation in adjuvant treatment selection, with many patients managed using endocrine-based strategies. These findings highlight the clinical relevance of integrating genomic and clinicopathological information in treatment decision-making and support the role of genomic assays as complementary tools for individualized therapy in this challenging population.

## Background

Adjuvant treatment decision-making for hormone receptor–positive (HR+), human epidermal growth factor receptor 2–negative (HER2-) breast cancer has been substantially refined by the incorporation of multigene assays. Among these, the 21-gene Oncotype DX Recurrence Score (RS) was originally developed to predict recurrence risk and chemotherapy benefit in node-negative disease and has since been widely adopted in clinical practice [1]. The TAILORx trial established that patients with low to intermediate RS could safely omit adjuvant chemotherapy without compromising survival, thereby positioning RS as a cornerstone of personalized adjuvant therapy [2].

The clinical utility of RS has subsequently been extended to patients with limited nodal involvement. In the RxPONDER trial, postmenopausal women with one to three positive lymph nodes and low RS derived no significant benefit from chemotherapy, whereas premenopausal women experienced improved invasive disease-free survival regardless of RS [3]. These findings highlighted the complexity of treatment decision-making in premenopausal, node-positive breast cancer and raised ongoing debate regarding the biological basis of chemotherapy benefit in this population.

In parallel with genomic assays, clinical risk stratification based on conventional clinicopathological factors remains central to therapeutic decision-making. International consensus guidelines, including the St. Gallen recommendations, integrate tumor size, nodal status, histological grade, and proliferative activity to define clinical risk [4]. Among these factors, Ki-67 has been repeatedly shown to be prognostically relevant, although optimal cut-off values remain controversial [5, 6].

The prognostic and predictive value of RS has also been demonstrated in node-positive disease, supporting its use beyond node-negative settings [7, 8]. Population-based and real-world studies further suggest that RS-guided treatment decisions are associated with favorable breast cancer–specific outcomes [9]. In contrast, other multigene assays, such as the 70-gene signature evaluated in the MINDACT trial, underscore the broader paradigm shift toward genomic-guided adjuvant therapy [10].

Despite these advances, discordance between clinical risk and genomic risk is frequently encountered in routine practice. Prospective and real-world studies have reported that a substantial proportion of patients classified as high risk based on clinicopathological criteria exhibit low genomic risk profiles [11, 12]. This discrepancy is particularly relevant given the increasing availability of targeted endocrine-based strategies, including CDK4/6 inhibitors and fluoropyrimidine-based regimens, which may offer alternatives to cytotoxic chemotherapy in selected patients [13–15].

Recent efforts have focused on integrating clinical and genomic information into composite models, such as RSClin, to refine risk stratification and treatment decisions [16, 17]. In parallel, trials evaluating adjuvant endocrine therapy intensification, including monarchE and POTENT, have expanded therapeutic options for patients with high clinical risk features [18, 19], while newer data on ribociclib further emphasize the evolving landscape of adjuvant systemic therapy [20].

Population-level analyses demonstrate that improvements in breast cancer mortality vary substantially by biological subtype, underscoring the importance of precision medicine approaches [21]. Contemporary guidelines increasingly emphasize the use of validated biomarkers to guide adjuvant therapy selection [22, 23], including optimal endocrine therapy strategies [24]. Nevertheless, technical and biological factors - such as concordance of RS between biopsy and surgical specimens [25] and discrepancies between clinical and genomic risk assessment [26] - continue to complicate treatment decisions. Moreover, emerging data on biologically distinct subgroups, such as estrogen receptor–low tumors, further highlight the heterogeneity underlying HR+ breast cancer [27].

Finally, while cytotoxic chemotherapy remains an important component of adjuvant therapy, large meta-analyses confirm that its benefit must be balanced against toxicity, particularly in biologically low-risk disease [28]. Real-world studies specifically examining RS-guided chemotherapy use in node-positive patients demonstrate substantial variation in treatment patterns, reflecting ongoing uncertainty in this setting [29, 30].

Against this background, the present study aimed to clarify the determinants of clinical–genomic risk discordance and its impact on real-world treatment selection, with a particular focus on premenopausal women with node-positive HR+/HER2- breast cancer.

## Methods

### Study design and patients

This retrospective cohort study was conducted at Osaka Metropolitan University Hospital. Consecutive patients with histologically confirmed HR+/HER2- breast cancer who underwent curative surgery and subsequent Oncotype DX testing between September 2023 and November 2025 were eligible for inclusion. Patients with invasive breast carcinoma who had complete clinicopathological data - including tumor size, histological grade, lymph node status, and Ki-67 index - and valid Oncotype DX RS results were included. Patients who received neoadjuvant systemic therapy, had recurrent or metastatic disease at diagnosis, or lacked complete clinicopathological or RS data were excluded. A total of 290 patients met the eligibility criteria and were included in the final analysis.

### Clinical risk assessment

Clinical risk (CR) was defined according to the St. Gallen International Consensus criteria [4]. Patients were classified as having high clinical risk if at least one of the following features was present: tumor size greater than 2 cm, lymph node metastasis, or histological grade 3 [4]. In addition, a Ki-67 index greater than 20% was used as a marker of high proliferative activity, in line with previous reports and real-world studies [5, 6]. Patients who did not meet any of these criteria were classified as low clinical risk. This definition is consistent with prior real-world studies evaluating clinical–genomic risk discordance in hormone receptor–positive, HER2-negative breast cancer [26].

### Oncotype DX testing and genomic risk classification

Oncotype DX testing was performed using the 21-gene reverse transcription polymerase chain reaction assay (Exact Sciences, Redwood City, CA, USA), as previously described [1]. The RS ranges from 0 to 100. Based on established clinical trials and institutional practice, genomic risk was categorized as high (RS ≥ 26) or low (RS < 26) [2, 3].

### Definition of concordance and discordance

Patients were categorized according to the concordance or discordance between clinical and genomic risk, consistent with prior reports [7, 26]. Concordant cases were defined as those with RS-high/CR-high or RS-low/CR-low profiles, whereas discordant cases were defined as RS-high/CR-low or RS-low/CR-high profiles.

### Treatment allocation

Adjuvant systemic therapy was determined by a multidisciplinary team in accordance with institutional and national guidelines [22–24]. Treatment strategies were categorized into three groups: (i) intravenous chemotherapy combined with endocrine therapy, (ii) endocrine therapy combined with systemic treatment intensification strategies (abemaciclib or S-1) based on patient risk profiles and contemporary evidence [18, 19], and (iii) standard endocrine therapy, defined as endocrine therapy with or without ovarian function suppression. Ovarian function suppression was considered part of the endocrine therapy backbone and was not used to define treatment intensity. Treatment distribution was evaluated in the overall cohort and specifically in a predefined subgroup of premenopausal patients with node-positive disease.

### Statistical analysis

Categorical variables were expressed as frequencies and percentages and compared using the chi-square test or Fisher’s exact test, as appropriate. To identify predictors of clinical–genomic discordance, univariate and multivariable binary logistic regression analyses were performed in the overall cohort and separately in patients with node-positive disease, as commonly applied in prior observational studies [26, 29]. Odds ratio (OR) and 95% confidence intervals (CI) were calculated. A two-sided *p* value < 0.05 was considered statistically significant. All statistical analyses were performed using JMP version 16 (SAS Institute, Cary, NC, USA).

### Ethical approval

This study was approved by the Institutional Review Board of Osaka Metropolitan University (approval number: #2025-045). The requirement for informed consent was waived due to the retrospective nature of the study and the use of anonymized data. The study was conducted in accordance with the principles of the Declaration of Helsinki.

## Results

### Patient characteristics and distribution of concordance status

A total of 290 patients with hormone receptor–positive, HER2-negative breast cancer who underwent Oncotype DX testing were included in the analysis. The distribution of concordance and discordance between CR and genomic risk according to menopausal and nodal status is summarized in Table 1. Overall, concordance was observed in 167 patients (57.6%), whereas discordance was identified in 123 patients (42.4%). Discordance was significantly more frequent in patients with node-positive disease than in those without nodal involvement (*p* = 0.034), and menopausal status showed a differential association with discordance depending on nodal status.

**Table 1:** Concordance/discordance by menopausal status in overall and nodal subgroups

	All			N0			N1		
Parameters	concordance(*n* = 167)	discordance(*n* = 123)	*p *value	concordance(*n* = 151)	discordance(*n* = 71)	*p *value	concordance(*n* = 16)	discordance(*n* = 52)	*p *value
Menopausal status Premenopausal Postmenopausal	41 (24.6%) 126 (75.4%)	45 (36.6%) 78 (63.4%)	0.027	38 (25.2%) 113 (74.8%)	20 (28.2%) 51 (71.8%)	0.635	3 (18.7%) 13 (81.3%)	25 (48.1%) 27 (51.9%)	0.034

### Predictors of clinical-genomic discordance in the overall cohort

Univariate and multivariable logistic regression analyses were performed to identify clinicopathological predictors of discordance in the overall cohort (Table 2). In univariate analysis, premenopausal status, larger tumor size (>20 mm), lymph node positivity, and Ki67 >20% were significantly associated with discordance. In multivariable analysis, tumor size greater than 20 mm (OR 8.327, 95% CI 4.440-15.617, *p* < 0.001) and node-positive status (OR 6.428, 95% CI 3.221-12.825, *p* < 0.001) remained independent predictors of discordance. These findings indicate that discordance between CR and RS is strongly associated with higher tumor burden in the overall population.

**Table 2:** Univariate and multivariate logistic regression analyses for predictors of discordance in the overall cohort

	Univariate analysis			Multivariate analysis		
Parameters	Odds ratio	95% CI	*p* value	Odds ratio	95% CI	*p* value
Age at examination (years) ≤ 50 vs. > 50	0.786	0.465–1.329	0.369			
Menopausal status Premenopausal vs. Postmenopausal	0.564	0.339–0.938	0.027	0.669	0.362–1.233	0.197
Tumor size (mm) > 20.0 vs. ≤ 20.0	8.596	4.816–15.341	< 0.001	8.327	4.440-15.617	< 0.001
Lymph node status Positive vs. Negative	6.912	3.691–12.943	< 0.001	6.428	3.221–12.825	< 0.001
Ki67 >20% vs. ≤ 20%	0.508	0.291–0.887	0.017	0.880	0.439–1.763	0.718
Histological grade Grade 3 vs. 1–2	0.927	0.414–2.075	0.853			
Progesterone receptor Negative vs. Positive	1.244	0.779–1.987	0.361			

*CI *confidence intervals

### Predictors of discordance in node-positive patients

To further clarify determinants of discordance in node-positive disease, logistic regression analyses were restricted to patients with lymph node metastasis (Table 3). In this subgroup, premenopausal status emerged as an independent factor inversely associated with discordance (OR 0.183, 95% CI 0.035–0.960, *p* = 0.045), whereas a high Ki67 index (>20%) was independently and positively associated with discordance (OR 4.814, 95% CI 1.013–22.879, *p* = 0.048). Tumor size and histological grade showed significant associations with discordance in univariate analyses; however, these associations were attenuated and did not remain significant after multivariable adjustment. Collectively, these findings suggest that, in node-positive disease, clinical–genomic discordance may reflect biological characteristics associated with premenopausal status, particularly proliferative activity.

**Table 3:** Univariate and multivariate logistic regression analyses for predictors of discordance in patients with node-positive disease

	Univariate analysis			Multivariate analysis		
Parameters	Odds ratio	95% CI	*p* value	Odds ratio	95% CI	*p* value
Age at examination (years old) ≤ 50 vs. > 50	0.436	0.110–1.731	0.238			
Menopausal status Premenopausal vs. Postmenopausal	0.249	0.063–0.979	0.047	0.183	0.035–0.960	0.045
Tumor size (mm) ≤ 20.0 vs. > 20.0	0.284	0.086–0.939	0.039	0.462	0.107–1.997	0.301
Ki67 >20% vs. ≤ 20%	7.000	2.057–23.823	0.002	4.814	1.013–22.879	0.048
Histological grade Grade 3 vs. 1–2	0.139	0.033–0.584	0.007	0.262	0.045–1.527	0.136
Progesterone receptor Negative vs. Positive	5.667	1.595–20.130	0.007	2.412	0.506–11.498	0.269

*CI* confidence intervals

### Distribution of clinical-genomic risk categories

**Fig. 1:**
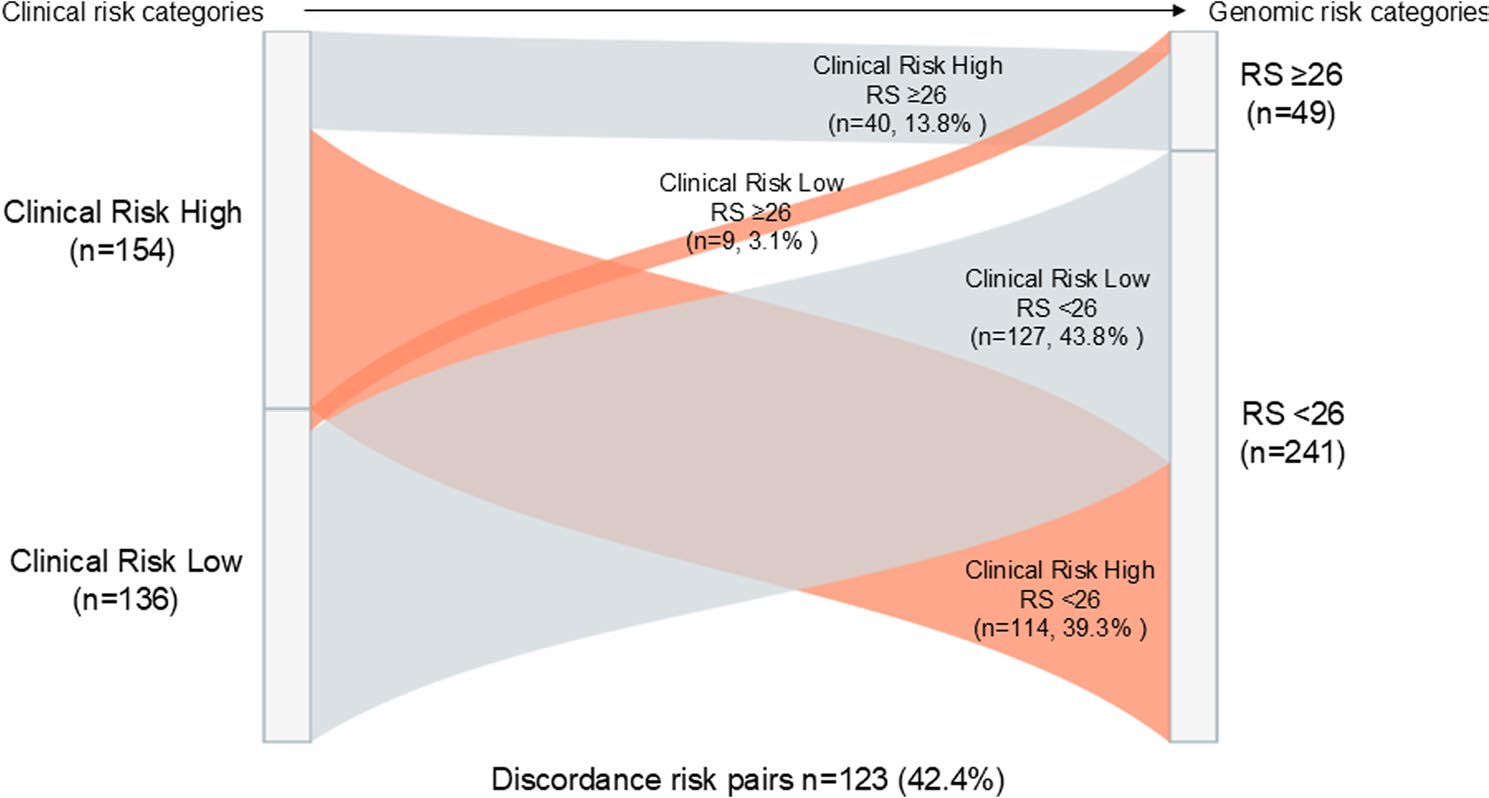
Distribution of clinical and genomic risk categories. Patients were stratified according to clinical risk (CR), defined by the St. Gallen criteria, and genomic risk based on the Oncotype DX Recurrence Score (RS). Genomic high risk was defined as RS ≥26. Concordant cases were defined as CR-high/RS-high and CR-low/RS-low, whereas discordant cases were defined as CR-high/RS-low or CR-low/RS-high. In the figure, concordant categories are shown in gray and discordant categories are shown in orange. Among the 290 patients analyzed, 49 patients (16.9%) were classified as RS-high, including 40 patients (13.8%) with high clinical risk and 9 patients (3.1%) with low clinical risk. In contrast, 241 patients (83.1%) had low genomic risk, including 127 patients (43.8%) with low clinical risk and 114 patients (39.3%) with high clinical risk. A substantial proportion of patients were therefore classified as clinically high risk despite having low genomic risk, indicating a substantial proportion of patients exhibited clinical–genomic discordance in this cohort

The distribution of clinical and genomic risk categories is shown in Fig. 1. Among the 290 patients analyzed, 49 patients (16.9%) were classified as RS-high (RS ≥26), including 40 patients (13.8%) with high clinical risk and 9 patients (3.1%) with low clinical risk. In contrast, 241 patients (83.1%) had low genomic risk (RS <26), including 127 patients (43.8%) with low clinical risk and 114 patients (39.3%) with high clinical risk. A substantial proportion of patients were therefore classified as clinically high risk despite having low genomic risk, highlighting the high prevalence of clinical–genomic discordance in this cohort.

### Treatment selection according to concordance status in premenopausal node-positive patients

**Fig. 2:**
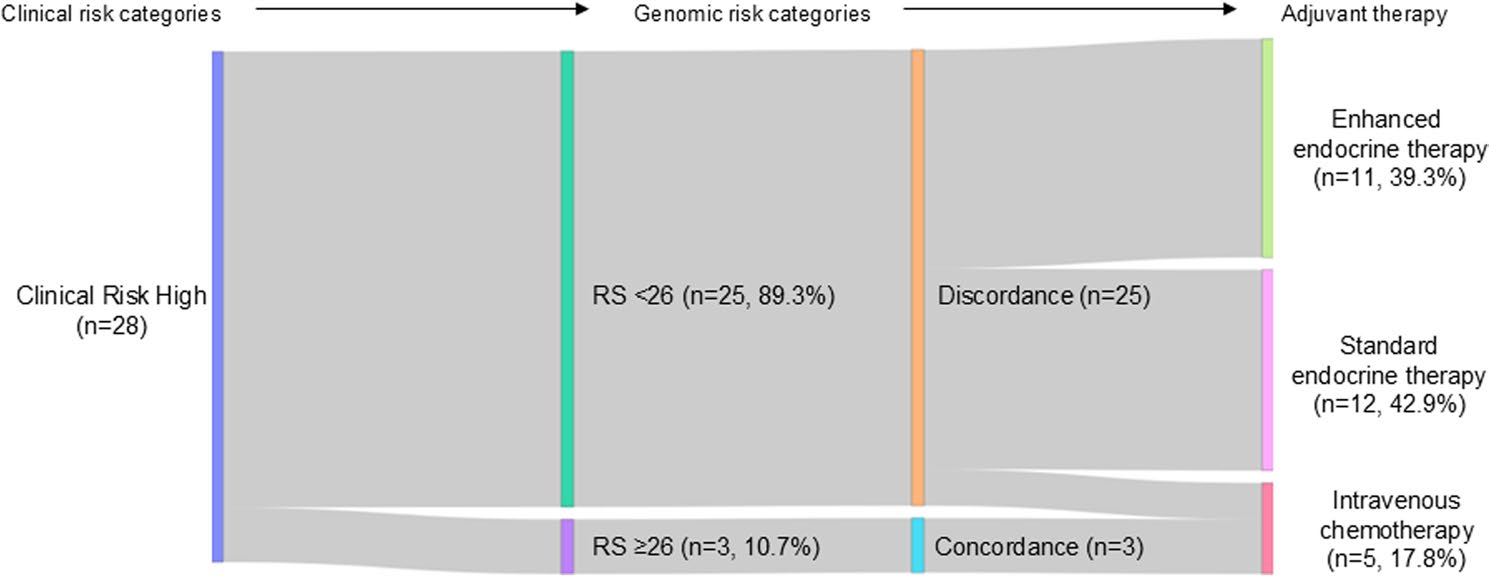
Treatment selection according to clinical–genomic concordance in premenopausal node-positive patients. Postoperative treatment strategies were compared according to concordance status between clinical and genomic risk in premenopausal patients with node-positive hormone receptor–positive, HER2-negative breast cancer. Treatments were categorized as intravenous chemotherapy, endocrine therapy combined with systemic treatment intensification (abemaciclib or S-1), or standard endocrine therapy (endocrine therapy with or without ovarian function suppression). Among concordant cases (*n* = 3), all patients received intravenous chemotherapy. In contrast, among discordant cases (*n* = 25), chemotherapy was administered in 2 patients (8%), enhanced endocrine therapy in 11 patients (44%), and standard endocrine therapy in 12 patients (48%). The distribution of treatment strategies differed significantly between concordant and discordant groups (*p* < 0.001)

Treatment allocation was further evaluated in a predefined subgroup of 28 premenopausal patients with node-positive disease (Table 4, Fig. 2). Among concordant cases (*n* = 3), all patients received intravenous chemotherapy. In contrast, discordant cases (*n* = 25) demonstrated variability in treatment selection: only 2 patients (8%) received intravenous chemotherapy, whereas 11 patients (44%) were treated with enhanced endocrine therapy and 12 patients (48%) received standard endocrine therapy alone. The distribution of postoperative treatment strategies differed significantly between concordant and discordant groups (*p* < 0.001).

**Table 4:** Distribution of Postoperative Treatment Strategies by Concordance Status in Premenopausal Patients with Node-Positive Breast Cancer

	Intravenous chemotherapy (*n* = 5)	Enhanced endocrine therapy (*n* = 11)	Standard endocrine therapy (*n* = 12)	*p* value
Discordance (*n* = 25)	2	11	12	
Concordance (*n* = 3)	3	0	0	< 0.001

Concordant = RS high/CR high or RS low/CR low; Discordant = RS high/CR low or RS low/CR high. Genomic risk was defined by Oncotype DX RS (High = RS ≥ 26). Clinical risk was based on St. Gallen criteria

*RS* Recurrence Score, *CR* Clinical risk

## Discussion

In this real-world cohort of patients with hormone receptor–positive, HER2-negative breast cancer, we demonstrated that discordance between clinical risk and genomic risk is common and clinically meaningful, particularly in premenopausal women with node-positive disease. Approximately 40% of patients exhibited discordance between St. Gallen–based clinical risk and Oncotype DX Recurrence Score, consistent with previous reports, but our findings extend prior work by identifying premenopausal-specific biological determinants and clarifying the impact of discordance on contemporary treatment selection [2, 9, 26, 29, 30].

In the overall cohort, discordance was strongly associated with tumor burden, as reflected by larger tumor size and nodal positivity. These results are in line with prior studies suggesting that conventional clinicopathological factors tend to classify patients with higher anatomical burden as high risk, even when genomic assays indicate a lower intrinsic recurrence risk [4–8]. This discrepancy underscores the conceptual difference between prognostic assessment based on tumor extent and that based on tumor biology [15].

Notably, when the analysis was restricted to node-positive patients, a distinct pattern emerged. Premenopausal status and high proliferative activity, as indicated by Ki-67 > 20%, were independently associated with discordance, whereas nodal status itself no longer retained significance. These findings suggest that, in premenopausal node-positive breast cancer, discordance is driven not merely by anatomical factors but by a unique biological context characterized by heightened proliferation and endocrine milieu [5, 6]. This observation is particularly relevant in light of the RxPONDER trial, which demonstrated a chemotherapy benefit in premenopausal women irrespective of RS, raising the possibility that ovarian function suppression rather than direct cytotoxic effects may account for much of the observed benefit [3].

Our analysis of treatment selection provides insight into how clinical–genomic discordance is associated with variability in real-world adjuvant treatment strategies. In premenopausal patients with node-positive disease, concordant high-risk cases consistently received chemotherapy, reflecting alignment between clinicopathological and genomic risk assessments. In contrast, discordant cases demonstrated substantial heterogeneity in postoperative treatment allocation, with many patients managed using endocrine-based approaches rather than cytotoxic chemotherapy. These findings suggest that genomic risk information is increasingly incorporated into multidisciplinary decision-making to individualize treatment intensity in routine clinical practice, even among patients traditionally considered to have high-risk disease based on nodal involvement alone [29, 30].

Importantly, the present study was not designed to evaluate the efficacy or safety of specific treatment strategies, nor to determine whether chemotherapy can be safely omitted in premenopausal node-positive patients with low genomic risk. Rather, our findings should be interpreted as descriptive observations of contemporary treatment patterns in a real-world setting. Although endocrine therapy intensification strategies, including the addition of abemaciclib or fluoropyrimidine-based regimens, have demonstrated clinical benefit in selected high-risk populations [13, 18, 19], the extent to which genomic risk assessment should guide treatment de-escalation in premenopausal node-positive disease remains uncertain. Randomized evidence indicates that chemotherapy may still confer clinically meaningful benefit in premenopausal patients regardless of genomic risk category, underscoring the need for cautious interpretation of observational data in this context [3, 16, 17].

Several methodological considerations further warrant attention. First, genomic testing in routine practice is often performed in patients with intermediate or uncertain clinicopathological risk profiles, which may introduce testing-selection bias and influence the observed proportion of clinical–genomic discordance. Therefore, the discordance rate reported in this cohort should be interpreted as reflecting patterns among tested patients rather than the entire population of hormone receptor–positive, HER2-negative breast cancer. Second, the number of premenopausal node-positive patients included in subgroup analyses was limited, resulting in small cell sizes and reduced statistical stability for treatment pattern comparisons. Third, the relatively short duration of follow-up precluded meaningful evaluation of survival outcomes or treatment benefit. Consequently, multivariable analyses in this setting should be regarded as exploratory and hypothesis-generating rather than definitive.

 Despite these limitations, the present findings provide clinically relevant real-world evidence that discordance between clinicopathological and genomic risk classifications represents a frequent and biologically meaningful phenomenon. Integrating genomic assay results with established clinical risk factors may help refine shared decision-making and support more individualized approaches to adjuvant therapy selection, particularly in complex scenarios such as premenopausal node-positive breast cancer [4, 22–24, 28].

## Conclusions

Clinical–genomic risk discordance is frequent in hormone receptor–positive, HER2-negative breast cancer and appears particularly relevant in premenopausal patients with node-positive disease. In this population, discordance was associated with distinct biological features, including high proliferative activity, and was linked to substantial variability in adjuvant treatment selection in real-world clinical practice. These findings underscore the clinical importance of integrating genomic and clinicopathological information when considering treatment strategies and support the role of genomic assays as complementary tools to facilitate individualized decision-making in this complex population.

## Data Availability

The datasets generated and analyzed during the current study are not publicly available due to institutional restrictions but are available from the corresponding author upon reasonable request.

## Abbreviations

BC: Breast Cancer
CI: Confidence Interval
CR: Clinical Risk
ER: Estrogen Receptor
HER2: Human Epidermal Growth Factor Receptor 2
HR: Hormone Receptor
IHC: Immunohistochemistry
Ki-67: Ki-67 Proliferation Index
OFS: Ovarian Function Suppression
OR: Odds Ratio
RS: Recurrence Score
RSClin: Recurrence Score–Clinical Integrated Model
TNBC: Triple-Negative Breast Cancer
